# Bacitracin for Injection Recall: Impact on Immediate Breast Implant Surgical Outcomes

**DOI:** 10.1155/2022/1389539

**Published:** 2022-08-31

**Authors:** Abigail R. Tirrell, Jenna C. Bekeny, Eshetu A. Tefera, David H. Song, Kenneth L. Fan

**Affiliations:** ^1^Division of Plastic and Reconstructive Surgery, Icahn School of Medicine at Mount Sinai, New York, NY, USA; ^2^Department of Plastic and Reconstructive Surgery, MedStar Georgetown University Hospital, Washington, District of Columbia, USA; ^3^Department of Biostatistics and Biomedical Informatics, MedStar Health Research Institute, Hyattsville, Maryland, USA

## Abstract

**Background:**

Triple-antibiotic irrigation of breast implant pockets is a mainstay of infection prophylaxis in breast reconstruction and augmentation. The recall of bacitracin for injection due to risk of anaphylaxis and nephrotoxicity in January 2020, a staple component of the irrigation solution, has raised concern for worsened postoperative sequelae. This study aimed to investigate pre- and post-recall implant-based breast surgery to analyze the impact of bacitracin in irrigation solutions on infection rates.

**Methods:**

All implant-based breast reconstruction or augmentation surgeries from January 2019 to February 2021 were retrospectively reviewed. In a regression discontinuity study design, patients were divided into pre- and post-recall groups. Patient demographics, surgical details, and outcomes including infection rates were collected. Differences in complication rates were compared between groups and with surgical and patient factors.

**Results:**

254 implants in 143 patients met inclusion criteria for this study, with 172 implants placed before recall and 82 placed after recall. Patients in each cohort did not differ in age, BMI, smoking status, or history of breast radiation or capsular contracture (*p* > 0.05). All breast pockets were irrigated with antibiotic solution, most commonly bacitracin, cefazolin, gentamycin, and povidone-iodine before recall (116,67.4%) and cefazolin, gentamycin, and povidone-iodine after recall (59,72.0%). There was no difference in incidence of infection (6.4% vs. 8.5%, *p*=0.551) or cellulitis (3.5% vs. 3.7%, *p*=0.959) before and after recall. Implant infection was associated with smoking history (*p* < 0.001) and increased surgical time (*p*=0.003).

**Conclusions:**

Despite the recent recall of bacitracin from inclusion in breast pocket irrigation solutions, our study demonstrated no detrimental impact on immediate complication rates. This shift in irrigation protocols calls for additional investigations into optimizing antibiotic combinations in solution, as bacitracin is no longer a viable option, to improve surgical outcomes and long-term benefits.

## 1. Introduction

Irrigation of breast implant pockets is a cornerstone for preventing infectious sequelae during implant-based breast reconstruction and augmentation. Postoperative infections of breast implant cases range between 1% and 35% [1]. Infections require antibiotic therapy, lead to undesired reoperations, and yield unsatisfactory results. For patients undergoing breast reconstruction after oncologic resection, postoperative infection is associated with delays in oncologic treatment [2]. Biofilm formation from inflammation and infections within the prosthetic pocket and capsule can contribute to subsequent capsular contracture (CC), a painful and distorting complication of breast implant surgery [1, 3].

Research has repeatedly demonstrated the benefits of antibiotic irrigation of breast pockets in reducing both bacterial load and rates of postoperative infection [4, 5]. Adams et al. explored the clinical efficacy of antibiotic irrigations, and their recommendation of a triple-antibiotic solution comprised of a cephalosporin, bacitracin, and gentamycin became the gold standard for intraoperative infection prevention in breast implant cases [4]. Even so, a consensus on optimal antibiotics to include has not been reached, and a variety of combinations is used among plastic surgeons in the United States [6]. The off-label use of povidone-iodine, an antiseptic, in irrigation has also been inconsistent [6]. Prior to 2000, povidone-iodine was commonly implemented as an irrigation material for breast implants; however, a moratorium on its use in irrigation was enacted by the US Food and Drug Administration (FDA) over concerns about silicone implant degradation [7]. Since its return to the market in 2017, it has been reintroduced to irrigation solutions by many plastic surgeons [6, 7].

On January 31, 2020, the FDA requested the withdrawal of bacitracin for injection after the Antimicrobial Drugs Advisory Committee deemed that its benefits were outweighed by its risks of nephrotoxicity and anaphylaxis [8, 9]. Though it has been used regularly for intraoperative irrigation in many surgical fields to prevent infection, it is now only approved for use in infant pneumonia and empyema [10]. Since bacitracin previously served as a staple antibiotic in breast implant irrigation solutions, concerns for worse postoperative outcomes have risen as a result of this recall.

Despite the potential for unfavorable results after the removal of bacitracin from irrigation solutions, this recall has provided an opportunity to reassess antimicrobial breast implant irrigation through a regression discontinuity experiment [11]. Therefore, the central aim of this study was to investigate outcomes after implant-based breast surgery before and after recall and analyze the impact bacitracin truly had on outcomes in the immediate postoperative period.

## 2. Methods

### 2.1. Study Design

All breast reconstruction or augmentation surgeries that involved the placement or exchange of silicone breast implants from January 2019 to February 2021 were retrospectively reviewed. Through a regression discontinuity study design, patients were divided into two groups based on the date of bacitracin for injection recall (January 31, 2020). Patients were excluded from analysis if their surgery involved placement of tissue expanders or revisional surgeries without implant exchange. A minimum period of six months of postoperative follow-up was required for inclusion in this analysis to allow for adequate time after the index surgery. This study was approved by the Institution Review Board (MHRI 2018-173).

### 2.2. Antimicrobial Surgical Technique

Surgical sites were prepared with 2% chlorhexidine gluconate in 70% isopropyl alcohol. All patients received standard systemic prophylaxis consisting of 2 grams of IV cefazolin unless an existing allergy required the use of an alternative antibiotic. Intraoperatively, all patients underwent antibiotic irrigation of the breast pocket(s). Gloves were changed prior to handling the breast implant, and an aseptic technique of implant placement was performed using a sterile surgical funnel. Implant manipulation after insertion into the breast pocket was minimized.

### 2.3. Data Collection

Demographic and comorbidity information including age, race, body mass index (BMI), and smoking history was collected. Charlson comorbidity index (CCI) scores were calculated to determine comorbidity burdens. Preoperative risk factors including a history of breast radiation, capsular contracture, or prior non-autologous reconstruction with an implant or tissue expander (TE) were recorded.

Details of surgical procedures were collected, including type of surgery. In patients who underwent reconstruction after oncologic resection, type of primary surgery, such as mastectomy or lumpectomy, was recorded. Reconstructive surgeries were also defined as immediate reconstruction with an implant, delayed-immediate reconstruction in which a TE was placed initially with subsequent exchange to an implant, and delayed reconstruction. Other details collected included surgical time, implant volume, use of acellular dermal matrix (ADM), and implant surgical plane. Type of irrigation solution was noted. The type and number of antibiotic and antiseptic additives were recorded. Surgical complications were collected, including infection, cellulitis, seroma, hematoma, and delayed healing. A follow-up period for each patient was determined.

### 2.4. Statistical Analysis

Continuous variables were described by means and standard deviations, and categorical variables were described by frequencies and percentages. Two-sample *t*-test was used to examine differences in the averages of continuous variables between groups when normality assumption was satisfied. Chi-squared test and Fisher's exact test compared differences in categorial frequencies between groups as appropriate. Bivariate analysis was performed to assess associations between complication rates and patient or surgical characteristics. Statistical analysis was performed using STATA v.15 (StataCorp, College Station, Texas) with significance defined as *p* ≤ 0.05.

## 3. Results

143 patients underwent breast augmentation or reconstruction surgery with 254 implants between January 2019 and February 2021. 172 implants were placed before recall in 97 patients, and 82 implants were placed after recall in 46 patients.

### 3.1. Patient Demographics, Comorbidities, and Breast History

Patient demographic and comorbidity characteristics are described in Table 1. The average patient age was 50.9 years (SD: 11.9) in the pre-recall group and 53.8 years (SD: 12.7) in the post-recall group (*p*=0.079). There was no difference between the two groups in CCI or patient-reported race (*p* > 0.05). Average patient BMI was 26.1 kg/m^2^ (SD: 5.1) and 26.3 kg/m^2^ (SD: 4.6) in the pre- and post-recall groups, respectively (*p*=0.662). Over 70% of patients had no history of smoking in both groups, with no statistically significant difference between groups (*p*=0.332).

Both groups had similar rates of implants in place prior to surgery (50.6% versus 54.9%; *p*=0.029); however, more patients had tissue expanders in place before recall in comparison with after recall (31.4% versus 17.1%; *p*=0.029) (Table 2). 16.1% of breasts operated on had a history of radiation therapy, with 29 (16.9%) in the pre-recall group and 12 (14.6%) in the post-recall group (*p*=0.652). A history of capsular contracture was present in 12.6% of breasts, with no difference between groups (*p*=0.080).

### 3.2. Surgical Details

Most surgeries were cancer reconstruction procedures, with 162 (94.2%) pre-recall procedures and 75 (91.5%) post-recall procedures involving implant-based oncologic reconstruction (*p*=0.417) (Table 3).  Table 4 specifies the details of oncologic surgery history and reconstruction timing in those patients. Pre-recall patients had more nipple-sparing mastectomies (NSM) (65.4% versus 50.7%) and less skin-sparing mastectomies (SSM) (34.7% versus 27.8%) when compared with post-recall ones (*p*=0.018). Other surgeries performed included simple mastectomies and modified radical mastectomies; only one lumpectomy was performed in each group. The two groups differed in reconstruction timing, with fewer immediate reconstructions (17.3% versus 30.7%) and more delayed-immediate reconstructions (29.6% versus 20.0%) in the pre-recall group (*p*=0.045).

In both groups, all incisions were either inframammary (IM) or transverse incisions. Additional surgical details are displayed in Table 3. All implants were silicone, and average implant volume did not differ before and after recall (452.4 mL versus 439.3 mL; *p*=0.497). There was no difference in implant plane between groups, with over 90% of implants placed in the pre-pectoral position (*p*=0.788). Use of ADM during reconstruction was more prevalent after recall, with 63.41% of implants placed with ADM after recall and 51.74% of implants before recall, though this difference was not statistically significant (*p*=0.08). Average surgical time was 122.7 minutes (SD: 66.6) before recall and 145.8 minutes (SD: 63.0) after recall (*p*=0.009).

### 3.3. Antimicrobial Irrigation Components

The most irrigation solution used was 0.9% normal saline (NS), with only two implants (1.2%) in the pre-recall group irrigated in lactated Ringer's solution (*p*=0.327) (Table 3). Before recall, most irrigation solutions were composed of triple-antibiotic solution (83.1%), while most post-recall solutions were double-antibiotic ones (82.9%) (*p* < 0.001). Antibiotics used before recall included bacitracin 50,000 units, cefazolin 1 gram, gentamycin sulfate 80 milligrams, vancomycin 1 gram, and polymyxin B 500,000 units in varying combinations according to patient allergies; the same antibiotics, except for bacitracin, were used after recall. Povidone-iodine 10% solution was used in solutions in 83.7% of pre-recall and 87.8% of post-recall surgeries.

Table 5 describes the antibiotic and antiseptic combinations added to irrigation before and after recall. The most common pre-recall composition was a triple-antibiotic solution of bacitracin, cefazolin, and gentamycin with povidone-iodine in 67.4% of implants; the same triple-antibiotic solution without povidone-iodine was used in 14.0% of implants. After recall, 72.0% of implants were irrigated with a double-antibiotic solution of cefazolin and gentamycin with povidone-iodine.

### 3.4. Surgical Complications

Complications are displayed in Table 6. The overall complication rate was 9.84%, with no difference between groups (9.88% versus 9.76%; *p*=0.952). Average implant infection and cellulitis rates were 7.1% and 3.5%, respectively, with no significant difference before and after recall (*p* > 0.05). Other complications included seroma in 2.0% of patients, hematoma in 1.2% of patients, and delayed healing in 0.8% of patients, with no differences between groups (*p*=0.329). Average follow-up period was 357.1 days (SD: 307.6) before recall and 168.5 days (SD: 119.0) after recall (*p* < 0.001).

Table 7 reveals complication rates specific to cases that utilized the gold standard triple-antibiotic irrigation solution before recall (bacitracin, cefazolin, gentamycin + povidone-iodine) and double-antibiotic solution after recall (cefazolin, gentamycin + povidone-iodine). There was no difference between pre- and post-recall cohorts in overall complication rate (16.38% versus 10.17%; *p*=0267) or infection rates (5.17% versus 8.47%; *p*=0.395). There was a significant decrease in other complications, including seroma, hematoma, and delayed healing (11.21% versus 1.69%; *p*=0.028) after the recall.

Patient and surgical factors were analyzed for association with complications in Table 8. Patient history of smoking, breast radiation, and increased surgical time was associated with the development of “any complication” (*p* < 0.05). Implant infection was associated with patient history of smoking, no history of prior prosthetic device, and increased surgical time (*p* > 0.05). Cellulitis was associated with longer surgical time (0.044).  Table 9 sub-stratified patient populations into smaller, more homogenous groups based on surgery type, history of radiation, use of ADM, prior prosthetic device, and reconstructive timing. With the exception of fewer overall complications in the cohort of patients with prior implant or TE after the recall (16.31% versus 3.39%; *p*=0.012), there was no change in complication rates after the recall in any patient subpopulation (*p* < 0.001).

## 4. Discussion

The use of topical antibiotics intraoperatively during implant-based breast augmentation has transpired for decades [12]. However, the popularity of triple-antibiotic irrigation, particularly cefazolin, gentamycin, and bacitracin, has become a mainstream option after substantial research [4]. The recent recall of bacitracin for injection, a critical component of this triple-antibiotic recipe, disrupts this archetype and brings concern for worsening postoperative complications.

This study assessed outcomes prior to and immediately after the bacitracin recall and demonstrated no detrimental impact on complication rates. Most implants placed at our institution prior to the recall were irrigated with triple-antibiotic solution in combination with povidone-iodine. Once bacitracin was removed from the irrigation protocol, there was no increase in overall complication, infection, cellulitis, or seroma rates. These results persisted when analyzing outcomes of cases that used only the gold standard triple-antibiotic combination of bacitracin, cefazolin, and gentamycin with povidone-iodine, before and after recall, as well as when patient subpopulations based on variations in surgery type and timing were analyzed for complication rates. Our early findings suggest that the recall of bacitracin from irrigation does not have a significant impact on immediate surgical outcomes, and thus alternative antibiotic and antiseptic sequences may be sufficient to provide thorough infection prophylaxis.

In addition to finding analogous outcomes after the bacitracin recall, this study also identified patient and surgical risk factors that were associated with postoperative complications. Patient history of smoking was associated with higher rates of infection, a well-known risk factor for sequelae particularly in breast reconstruction [1]. Our data also revealed that lengthier surgical times were correlated with higher complication rates, including infection and cellulitis. While longer times increase exposure to pathogens, there was no difference between pre- and post-recall groups despite longer times after recall.

Irrigation with triple antibiotics has become the standard of care; however, a survey of American Society of Plastic Surgery (ASPS) surgeons in 2018 found a lack of consensus with over 30 irrigation solution combinations in use [6]. Triple-antibiotic solution without povidone-iodine was favored by over 40% of members, with an additional 17% using triple-antibiotic solution with povidone-iodine [6]. Other surgeons use differing numbers of antibiotics or singularly dilute povidone-iodine solution [6]. Antibiotic selection aims to cover a broad spectrum of organisms; more than 75% of implant infections are Gram-positive and typically involve *Staphylococcus aureus* and *Staphylococcus epidermidis*; however, many are Gram-negative consisting of *Pseudomonas* and *Escherichia coli* [13]. Bacitracin was commonly used due to its low cost, broad spectrum of activity, and high potency versus Gram-positive skin flora [13]. Gentamycin has Gram-negative coverage including *Pseudomonas* while cefazolin is broad spectrum with good *Staphylococcus* coverage; thus, organisms covered by bacitracin should also be covered by the remaining two antibiotics [13]. Additionally, *in vitro* studies have shown that the combination of 10% povidone-iodine, gentamycin, and cefazolin is completely effective at eradicating strains of *S. aureus*, *S. epidermidis, Pseudomonas,* and *E. coli* [14].

Prior studies of the use of these antibiotics in irrigation solution have shown major reductions in infection rates in comparison with normal saline solution [13]. Even single antibiotic solutions have protective benefits against postoperative infections and reduced rates of other complications such as seromas [15, 16]. Three antibiotics, however, may reduce infection rates substantially more. Adams et al. found infection rates less than 1% with triple-antibiotic irrigation [4]. Whether or not to combine triple-antibiotic irrigation with antiseptics such as povidone-iodine has been inconclusive as to whether it results in superior infection rates [5]. *In vitro* studies revealed that it reduces bacterial load by a factor of 10^4^ to 10^5^ for all methicillin-sensitive and resistant *S. aureus* and *S. epidermidis* strains and vancomycin-resistant *Enterococcus*, more effectively than triple-antibiotic solution is able to [7, 13]. Despite clinical data lacking comprehensive evidence for its combination with triple-antibiotic solution, povidone-iodine was used in more than 85% of implant cases in this study, which highlights its potential benefits in preventing infections.

Capsular contracture is an intricate complication potentiated by tissue trauma, bacteria, and other sources of local inflammation; breast pocket irrigation, with or without antibiotics, is universally practiced as prophylaxis [4]. Conflicting data exists regarding the true impact of antibiotic irrigation on capsular contracture. A study by Giordano et al. found that triple-antibiotic irrigation plus povidone-iodine resulted in a capsular contracture rate of 0.6% in comparison to 6.0% without irrigation [17]. Another study found the rate of grade III-IV capsular contracture to be only 1.8% with triple-antibiotic solution [4]. These findings were opposed by a systematic review by Samargandi et al., which identified only one randomized controlled trial and a few non-randomized studies that provided weak evidence for antibiotic irrigation as contracture prevention [18]. Despite the limited evidence, it is outweighed by the benefits of infection prevention and therefore is used regularly in implant surgery [19]. Although this study did not have sufficient follow-up to perform a true analysis of capsular contracture rates and their relationship to operative antibiotic solutions, future studies should investigate this dynamic.

Strengths of this study lie in its design as a regression discontinuity quasi-experiment. Regression discontinuity design allows patients to be assigned to intervention versus control groups based on falling above or below an arbitrary cutoff, in this case the date of bacitracin for injection recall [11]. Data on each side of the discontinuation are otherwise similar; there is no crossover of treatments nor nonrandom attrition of patients, and behavioral phenomena occur equally on both sides [11]. Patient cohorts were similar before and after recall in terms of surgery types, comorbidities, reconstruction timing, history of radiation or capsular contracture, and even incisional approach, as inframammary and transverse incisions have lower rates of infection in general in comparison with periareolar incisions [3].

Despite these advantages, this study is limited overall by its sample size and follow-up period. Though all cases involved implant-based breast surgery, there were variations in reconstructive timing, use of ADM, and prior prosthetic device use that introduce confounding factors; our sub-analyses of our patient population suggest consistent results before and after recall in these groups; however, larger studies of these individual cohorts will need to be pursued to fully understand the impact of the recall. There was also a substantially shorter follow-up period after recall, being likely the result of the temporal change in bacitracin use in combination with environmental factors. Briefly after the bacitracin recall, the COVID-19 pandemic started in March 2020, which significantly altered patient care towards virtual visits; patients may have avoided returning for follow-up, and those that engaged in telemedicine may not have caught early findings of various complications [20]. Additionally, while the bacitracin recall was the only major change in surgical protocols, causal inference in regression discontinuity study designs may be impacted by nonrandom manipulation around the treatment cutoff, such the COVID-19 pandemic [11].

Optimizing outcomes after breast implant surgery will call for larger studies to assess different antibiotic and antiseptic combinations, as the gold standard recipe with bacitracin is no longer an option. This also allows us to reevaluate antibiotic use, so that it is used judiciously without unnecessary additives, preventing the spread of drug resistance among pathogens [9]. Among the antibiotics used, Gram-positive and Gram-negative coverage must be achieved, as with cefazolin and gentamycin alone [13]. This study provides reassurance that bacitracin in triple-antibiotic solutions may not have been the keystone antibiotic required to quell most complications. Additional investigation of the long-term development of capsular contracture before and after recall, however, is necessary to fully elucidate the impact of the recall. Further, the consistent inclusion of povidone-iodine should be explored. After the 2000 moratorium on povidone-iodine by the FDA out of concern for silicone degradation, triple-antibiotic irrigation without antiseptics became the model [7]. After its return to irrigation in 2017, a large segment of surgeons still favored antibiotic solution without povidone-iodine [6, 13]. Its use as a primary irrigation should be reexplored given the known prophylactic effects on infection rates.

## 5. Conclusions

The recall of bacitracin for injection from use in intraoperative irrigation in January 2020 did not lead to worsened immediate complication rates after implant-based breast reconstruction and augmentation. This major shift in antibiotic irrigation protocols calls for additional investigation into the gold standard of preventative pocket irrigation, as bacitracin is no longer a viable option. Continued advancements in antimicrobial stewardship, in combination with optimizing surgical techniques, will improve surgical outcomes, patient satisfaction, and long-term benefits after breast implant surgery.

## Figures and Tables

**Table 1:** Patient demographics and comorbidities.

	Total (*n* = 254)	Pre-recall (*n* = 172)	Post-recall (*n* = 82)	*p* value
Age, years	51.83 ± 12.20	50.90 ± 11.86	53.78 ± 12.73	0.079
BMI, kg/m^2^	26.14 ± 4.94	26.05 ± 5.10	26.34 ± 4.61	0.662
CCI	1.48 ± 1.00	1.49 ± 1.05	1.45 ± 0.90	0.783
Smoking history				
Never smoker	187 (73.62)	124 (72.09)	63 (76.83)	0.332
Former smoker	63 (24.80)	44 (25.58)	19 (23.17)	
Current smoker	4 (1.57)	4 (2.33)	0 (0.00)	
Race/ethnicity				
White	152 (59.84)	100 (58.14)	52 (63.41)	0.143
African American	67 (26.38)	52 (30.23)	15 (18.29)	
Asian	16 (6.30)	10 (5.81)	6 (7.32)	
Other	19 (7.48)	10 (5.81)	9 (10.98)	

BMI: body mass index, CCI: Charlson comorbidity index. Bold values indicate statistical significance.

**Table 2:** Breast history.

	Total (*n* = 254)	Pre-recall (*n* = 172)	Post-recall (*n* = 82)	*p* value
History of breast radiation therapy	41 (16.14)	29 (16.86)	12 (14.63)	0.652
History of capsular contracture	32 (12.60)	26 (15.12)	6 (7.32)	0.080
Prior prosthetic device				
Implant	132 (51.97)	87 (50.58)	45 (54.88)	**0.029**
Tissue expander	68 (26.77)	54 (31.40)	14 (17.07)	
None	54 (21.26)	31 (18.02)	23 (23.05)	

Bold values indicate statistical significance.

**Table 3:** Surgical details.

	Total (*n* = 254)	Pre-recall (*n* = 172)	Post-recall (*n* = 82)	*p* value
Surgery type				
Augmentation	17 (6.69)	10 (5.81)	7 (8.54)	0.417
Cancer reconstruction	237 (93.31)	162 (94.19)	75 (91.46)	
Surgical time	130.11 ± 66.19	122.71 ± 66.57	145.84 ± 62.95	**0.009**
Implant volume	448.19 ± 143.16	452.41 ± 140.80	439.32 ± 148.48	0.497
Use of acellular dermal matrix	141 (55.51)	89 (51.74)	52 (63.41)	0.080
Implant surgical plane				
Pre-pectoral	231 (90.94)	157 (91.28)	74 (90.24)	0.788
Retro-pectoral	23 (9.06)	15 (8.72)	8 (9.76)	
Irrigation solution				
0.9% Normal saline	252 (99.21)	170 (98.84)	82 (100.00)	0.327
Lactated Ringer's	2 (0.79)	2 (1.16)	0 (0.00)	
Number of antibiotic additives				
1	11 (4.33)	5 (2.91)	6 (7.32)	**<0.001**
2	92 (36.22)	24 (13.95)	68 (82.93)	
3	151 (59.45)	143 (83.14)	8 (9.76)	
Antibiotics used				
Bacitracin	172 (67.72)	172 (100.00)	0 (0.00)	**<0.001**
Cefazolin	212 (83.46)	142 (82.56)	70 (85.37)	0.573
Gentamycin	245 (96.46)	165 (95.93)	80 (97)	0.511
Vancomycin	8 (3.15)	2 (1.16)	6 (7.32)	0.009
Polymyxin B	10 (3.94)	0 (0.00)	10 (12.20)	**<0.001**
Povidone-iodine 10%	216 (85.04)	144 (83.72)	72 (87.80)	0.394

Bold values indicate statistical significance.

**Table 4:** Oncologic reconstruction details.

	Total (*n* = 237)	Pre-recall (*n* = 162)	Post-recall (*n* = 75)	*p* value
Breast cancer surgery				
Nipple-sparing mastectomy	144 (60.76)	106 (65.43)	38 (50.67)	**0.018**
Skin-sparing mastectomy	71 (29.96)	45 (27.78)	26 (34.67)	
Simple mastectomy	8 (3.38)	2 (1.23)	6 (8.00)	
Modified radical mastectomy	8 (3.38)	6 (3.70)	2 (2.67)	
Radical mastectomy	2 (0.84)	0 (0.00)	2 (2.67)	
Total mastectomy	2 (0.84)	2 (1.23)	0 (0.00)	
Lumpectomy	2 (0.84)	1 (0.62)	1 (1.33)	
Reconstruction timing				
Immediate	51 (21.52)	28 (17.28)	23 (30.67)	**0.045**
Delayed-immediate	63 (26.58)	48 (29.63)	15 (20.00)	
Delayed	123 (51.90)	86 (53.09)	37 (49.33)	

Bold values indicate statistical significance.

**Table 5:** Antibiotic and antiseptic irrigation components.

Irrigation components	Implants irrigated
Pre-recall (*n* = 172)	
Bacitracin, cefazolin, gentamycin + povidone-iodine	116 (67.44)
Bacitracin, cefazolin, gentamycin	24 (13.95)
Bacitracin, gentamycin + povidone-iodine	22 (12.79)
Bacitracin	4 (2.33)
Bacitracin, gentamycin, vancomycin + povidone-iodine	2 (1.16)
Bacitracin, cefazolin + povidone-iodine	2 (1.16)
Bacitracin + povidone-iodine	2 (1.16)
Post-Recall (*n* = 82)	
Cefazolin, gentamycin + povidone-iodine	59 (71.95)
Cefazolin, gentamycin, polymyxin B + povidone-iodine	5 (6.10)
Gentamycin, vancomycin + povidone-iodine	4 (4.88)
Cefazolin, gentamycin	3 (3.66)
Gentamycin, vancomycin, polymyxin B	2 (2.44)
Gentamycin, polymyxin B + povidone-iodine	2 (2.44)
Gentamycin + povidone-iodine	2 (2.44)
Cefazolin	2 (2.44)
Gentamycin	2 (2.44)
Cefazolin, gentamycin, polymyxin B	1 (1.22)

Bold values indicate statistical significance.

**Table 6:** Pre- and post-recall outcomes.

	Total (*n* = 254)	Pre-recall (*n* = 172)	Post-recall (*n* = 82)	*p* value
Complications				
Any complication	25 (9.84)	17 (9.88)	8 (9.76)	0.952
Infection	18 (7.09)	11 (6.40)	7 (8.54)	0.551
Cellulitis	9 (3.54)	6 (3.49)	3 (3.66)	0.959
Other complications	10 (3.94)	6 (3.49)	4 (4.88)	0.329
Seroma	5 (1.97)	2 (1.16)	3 (3.66)	
Hematoma	3 (1.18)	2 (1.16)	1 (1.22)	
Delayed healing	2 (0.79)	2 (1.16)	0 (0.00)	
Follow-up period, days	296.22 ± 276.23	357.10 ± 307.64	168.51 ± 118.96	**<0.001**

Bold values indicate statistical significance.

**Table 7:** Complication rates with gold standard antibiotic combinations^*∗*^.

	Total (*n* = 175)	Pre-recall (*n* = 116)	Post-recall (*n* = 59)	*p* value
Complications				
Any complication	25 (14.29)	19 (16.38)	6 (10.17)	0.267
Infection	11 (6.29)	6 (5.17)	5 (8.47)	0.395
Cellulitis	5 (2.86)	4 (3.45)	1 (1.69)	0.510
Other Complications	14 (8.00)	13 (11.21)	1 (1.69)	**0.028**

^*∗*^Gold standard antibiotic combinations indicate the use of bacitracin + cefazolin + gentamycin with povidone-iodine before recall, and cefazolin + gentamycin with povidone-iodine after recall. Bold values indicate statistical significance.

**Table 8:** Outcomes by patient and surgical factors.

	Complication		
	Yes	No	*p* value
Any complication (*n* = 25)			
Age	54.64 ± 13.35	51.56 ± 12.10	0.234
BMI	27.96 ± 5.92	25.99 ± 4.78	0.059
CCI	1.60 ± 0.87	1.48 ± 1.01	0.555
Smoking history			
Never smoker	16 (64.00)	169 (74.45)	**<0.001**
Former smoker	6 (24.00)	57 (25.11)	
Current smoker	3 (12.00)	1 (0.44)	
History of breast radiation therapy	8 (32.00)	33 (14.54)	**0.025**
History of capsular contracture	5 (20.00)	25 (11.01)	0.188
Prior prosthetic device			
Tissue expander	6 (24.00)	62 (27.31)	0.053
Implant	9 (36.00)	121 (53.30)	
None	10 (40.00)	44 (19.38)	
Surgery type			
Augmentation	0 (0.00)	17 (7.49)	0.157
Reconstruction	25 (100.00)	210 (92.51)	
Total surgical time	186.96 ± 97.68	124.25 ± 58.96	**<0.001**
Implant volume	498.20 ± 140.76	441.78 ± 142.94	0.062
Implant surgical plane			
Pre-Pectoral	24 (96.00)	205 (90.31)	0.348
Retro-pectoral	1 (4.00)	22 (9.69)	
Use of acellular dermal matrix	17 (68.00)	124 (54.63)	0.201
Number of antibiotics	2.44 ± 0.65	2.57 ± 0.57	0.294
Povidone-iodine 10%	20 (80.00)	194 (95.46)	0.469
Infection (*n* = 18)			
Age	54.58 ± 14.93	51.62 ± 11.98	0.322
BMI	28.48 ± 6.04	25.96 ± 4.81	**0.037**
CCI	1.56 ± 0.86	1.47 ± 1.01	0.728
Smoking history			
Never smoker	10 (55.56)	177 (75.00)	**<0.001**
Former smoker	5 (27.78)	58 (24.58)	
Current smoker	3 (16.67)	1 (0.42)	
History of breast radiation therapy	5 (27.78)	36 (15.25)	0.164
History of capsular contracture	2 (1.11)	30 (12.71)	0.844
Prior prosthetic device			
Tissue expander	4 (22.22)	64 (27.12)	**0.007**
Implant	5 (27.78)	127 (53.81)	
None	9 (50.00)	45 (19.07)	
Surgery type			
Augmentation	0 (0.00)	17 (7.20)	0.238
Reconstruction	18 (100.00)	219 (92.80)	
Total surgical time	174.72 ± 87.97	126.70 ± 63.18	**0.003**
Implant volume	510.56 ± 120.26	443.43 ± 143.87	0.055
Implant surgical plane			
Pre-pectoral	17 (94.44)	214 (90.68)	0.591
Retro-pectoral	1 (5.56)	22 (9.32)	
Use of acellular dermal matrix	12 (66.67)	129 (54.66)	0.323
Number of antibiotics	2.33 ± 0.69	2.57 ± 0.57	0.098
Povidone-iodine 10%	14 (37.84)	202 (93.09)	0.370
Cellulitis (*n* = 9)			
Age	52.94 ± 13.35	51.79 ± 12.18	0.781
BMI	23.85 ± 2.64	26.22 ± 4.99	0.157
CCI	1.33 ± 1.00	1.48 ± 1.00	0.663
Smoking history			
Never smoker	6 (66.67)	181 (73.88)	0.786
Former smoker	3 (33.33)	60 (24.49)	
Current smoker	0 (0.00)	4 (1.63)	
History of breast radiation therapy	3 (33.33)	38 (15.51)	0.153
History of capsular contracture	2 (22.22)	30 (12.24)	0.376
Prior prosthetic device			
Tissue expander	1 (1.11)	67 (27.35)	0.191
Implant	4 (4.44)	128 (52.24)	
None	4 (4.44)	50 (20.41)	
Surgery type			
Augmentation	0 (0.00)	17 (6.94)	0.413
Reconstruction	9 (100.00)	228 (93.06)	
Total surgical time	173.67 ± 85.31	128.51 ± 65.05	**0.044**
Implant volume	477.78 ± 155.85	447.10 ± 142.91	0.529
Implant surgical plane			
Pre-pectoral	9 (100.00)	222 (90.61)	0.335
Retro-pectoral	0 (0.00)	23 (9.39)	
Use of acellular dermal matrix	5 (55.56)	136 (55.51)	0.998
Number of antibiotics	2.56 ± 0.53	2.55 ± 0.58	0.982
Povidone-iodine 10%	8 (88.89)	208 (84.90)	0.742

BMI: body mass index, CCI: Charlson comorbidity index. Bold values indicate statistical significance.

**Table 9:** Sub-stratification of case outcomes.

	Total	Pre-recall	Post-recall	*p* value
Surgery type				
Reconstructive	*n* = 237	*n* = 162	*n* = 75	
Number of antibiotics	2.53 ± 0.59	2.79 ± 0.48	1.97 ± 0.37	**<0.001**
Complications				
Any complication	37 (15.62)	28 (17.28)	9 (12.00)	0.297
Infection	18 (7.59)	11 (6.79)	7 (9.33)	0.492
Cellulitis	9 (3.80)	6 (3.70)	3 (4.00)	0.912
Other complications	10 (4.22)	6 (3.70)	4 (5.33)	0.562
Augmentation	*n* = 17	*n* = 10	*n* = 7	
Number of antibiotics	2.82 ± 0.39	3.00 ± 0	2.57 ± 0.53	0.103
Complications				
Any complication	0 (0.00)	0 (0.00)	0 (0.00)	1.0
Infection	0 (0.00)	0 (0.00)	0 (0.00)	1.0
Cellulitis	0 (0.00)	0 (0.00)	0 (0.00)	1.0
Other complications	0 (0.00)	0 (0.00)	0 (0.00)	1.0
History of breast radiation				
Prior RT	*n* = 34	*n* = 26	*n* = 8	
Number of antibiotics	2.65 ± 0.49	2.84 ± 0.38	2.00 ± 0.00	**<0.001**
Complications				
Any complication	12 (35.29)	11 (42.31)	1 (12.50)	0.123
Infection	4 (11.76)	3 (11.54)	1 (12.50)	0.941
Cellulitis	2 (5.88)	2 (7.69)	0 (0.00)	0.419
Other complications	3 (8.82)	3 (11.54)	0 (0.00)	0.314
No prior RT	*n* = 208	*n* = 134	*n* = 74	
Number of antibiotics	2.51 ± 0.60	2.78 ± 0.50	2.03 ± 0.44	**<0.001**
Complications				
Any complication	25 (12.02)	17 (12.69)	8 (10.81)	0.690
Infection	14 (6.73)	8 (5.97)	6 (8.11)	0.556
Cellulitis	7 (3.37)	4 (2.99)	3 (4.05)	0.682
Other complications	7 (3.37)	3 (2.24)	4 (5.41)	0.225
Use of acellular dermal matrix				
ADM used	*n* = 86	*n* = 49	*n* = 37	
Number of antibiotics	2.48 ± 0.55	2.80 ± 0.41	2.05 ± 040	**<0.001**
Complications				
Any complication	15 (17.44)	7 (14.29)	8 (21.62)	0.375
Infection	9 (10.47)	3 (6.12)	6 (16.22)	0.130
Cellulitis	5 (5.81)	2 (4.08)	3 (8.11)	0.430
Other complications	6 (6.98)	2 (4.08)	4 (10.81)	0.225
ADM not used	*n* = 168	*n* = 123	*n* = 45	
Number of antibiotics	2.59 ± 0.59	2.80 ± 0.49	2.00 ± 0.43	**<0.001**
Complications				
Any complication	22 (13.10)	21 (17.07)	1 ()	**0.012**
Infection	9 (5.36)	8 (6.50)	1 ()	0.275
Cellulitis	4 (2.38)	4 (3.25)	0 (0.00)	0.221
Other complications	4 (2.38)	4 (3.25)	0 (0.00)	0.221
Reconstruction timing				
Immediate	*n* = 51	*n* = 28	*n* = 23	
Number of antibiotics	2.57 ± 0.50	2.89 ± 0.31	2.17 ± 0.39	**<0.001**
Complications				
Any complication	13 (25.50)	6 (21.43)	7 (30.43)	0.463
Infection	10 (19.61)	4 (14.29)	6 (26.09)	0.291
Cellulitis	5 (9.80)	2 (7.14)	3 (13.0)	0.481
Other complications	4 (7.84)	1 (3.57)	3 ()	0.211
Delayed-immediate	*n* = 63	*n* = 48	*n* = 15	
Number of antibiotics	2.56 ± 0.62	2.77 ± 0.52	1.87 ± 0.35	**<0.001**
Complications				
Any complication	9 ()	9 ()	0 ()	0.070
Infection	3 ()	3 ()	0 ()	0.321
Cellulitis	0 ()	0 ()	0 ()	1.0
Other complications	2 ()	2 ()	0 ()	0.422
Delayed	*n* = 123	*n* = 86	*n* = 37	
Number of antibiotics	2.50 ± 0.61	2.77 ± 0.50	1.89 ± 0.31	**<0.001**
Complications				
Any complication	15 ()	13 ()	2 ()	0.131
Infection	5 ()	4 ()	1 ()	0.616
Cellulitis	4 ()	4 ()	0 ()	0.182
Other complications	4 ()	3 ()	1 ()	0.822
History of prosthetic device				
Prior implant or TE	*n* = 200	*n* = 141	*n* = 59	
Number of antibiotics	2.54 ± 0.60	2.78 ± 0.49	1.97 ± 0.41	**<0.001**
Complications				
Any complication	25 (12.50)	23 (16.31)	2 (3.39)	**0.012**
Infection	9 (4.50)	8 (5.67)	1 (1.69)	0.216
Cellulitis	5 (2.50)	5 (3.55)	0 (0.00)	0.143
Other complications	6 (3.00)	5 (3.55)	1 (1.69)	0.484
No prior device	*n* = 54	*n* = 31	*n* = 23	
Number of antibiotics	2.59 ± 0.50	2.90 ± 0.30	2.17 ± 0.39	**<0.001**
Complications				
Any complication	12 (2.22)	5 (16.13)	7 (30.43)	0.211
Infection	9 (16.67)	3 (9.68)	6 (26.09)	0.110
Cellulitis	4 (7.41)	1 (3.23)	3 (13.04)	0.173
Other complications	4 (7.41)	1 (3.23)	3 (13.04)	0.173

RT: radiation therapy, ADM: acellular dermal matrix, TE: tissue expander. Bold values indicate statistical significance.

## Data Availability

The clinical data used to support the findings of this study can be obtained from the corresponding author upon request.
